# Quality of Life and Toxicities Following Whole-Palm Versus Involved-Field Radiation Therapy for Dupuytren’s Disease: A Substudy From a Randomized Trial

**DOI:** 10.1016/j.adro.2026.102083

**Published:** 2026-09-11

**Authors:** Renee Duvenage, Jarad M. Martin, Tanya Burgess, Brett McClelland, David Christie, Paul M.N. Werker, Warren Rozen, Roel J.H.M. Steenbakkers, Anneke de Haan, Sreelakshmi Anoora, Dion Sandoz, Joshua Sappiatzer, Katherine Neville, Sandy Sampaio, David Schlect, Joseph Bucci, David Hunter-Smith, David Dilley

**Affiliations:** a GenesisCare Gateshead, Newcastle, New South Wales, Australia; b School of Medicine and Public Health, University of Newcastle, Newcastle, New South Wales, Australia; c Hunter Hand Surgery, Newcastle, New South Wales, Australia; d GenesisCare Tugun, Queensland, Australia; e Bond University, Gold Coast, Queensland, Australia; f Department of Plastic Surgery, University Medical Center Groningen, University of Groningen, Groningen, The Netherlands; g Monash University, Melbourne, Victoria, Australia; h Department of Radiation Oncology, University Medical Center Groningen, University of Groningen, Groningen, The Netherlands; i GenesisCare Research, Sydney, New South Wales, Australia; j Hunter Hand Rehabilitation Centre, Newcastle, New South Wales, Australia; k GenesisCare Adelaide, Adelaide, South Australia, Australia; l GenesisCare Hurstville, Sydney, New South Wales, Australia; m GenesisCare Auchenflower, Queensland, Australia; n GenesisCare Sydney, Sydney, New South Wales, Australia; o Dilley Practice, Sydney, New South Wales, Australia; p Department of Radiation Oncology, Calvary Mater Newcastle, Newcastle, New South Wales, Australia

## Abstract

**Purpose:**

The Dupuytren’s disease Evaluation of Preventative or Adjuvant Radiation Therapy trial compared low-dose radiation therapy (LDRT) with observation for contracture prevention in early Dupuytren’s disease. Guidelines vary regarding field selection due to concerns about toxicity in larger fields. In the LDRT arm of the prevention group, the permitted treatment fields were either whole-palm radiation therapy (WPRT) or involved-field radiation therapy (IFRT). This substudy compares quality-of-life and toxicity outcomes between WPRT and IFRT.

**Methods and Materials:**

This substudy analyzed 202 patients (202 treated hands) treated with LDRT (30 Gy in 10 fractions, split-course) in the Dupuytren’s disease Evaluation of Preventative or Adjuvant Radiation Therapy trial prevention cohort. Quality of life was assessed using the pain intensity visual analog scale, Quick Disabilities of the Arm, Shoulder, and Hand, and Unité Rhumatologique des Affections de la Main at baseline and at 6, 12, and 36 months post-treatment. Toxicities were graded according to the National Cancer Institute Common Terminology Criteria for Adverse Events, version 4.03.

**Results:**

Of 202 hands treated, 126 (62.4%) received WPRT and 76 (37.6%) received IFRT. Baseline characteristics were balanced between the groups. The pain intensity visual analog scale, Quick Disabilities of the Arm, Shoulder, and Hand, and Unité Rhumatologique des Affections de la Main scores did not differ significantly between the groups at any time point. LDRT was well tolerated with no grade 3 or higher toxicity observed. Acute toxicity was low grade, as expected, and more frequently reported following WPRT than IFRT. Persistent late effects were uncommon, with a <5% incidence of low-grade reduced sweating in the WPRT cohort.

**Conclusions:**

In early Dupuytren’s disease, LDRT delivered using either WPRT or IFRT was well tolerated and associated with comparable patient-reported outcomes through 36 months. Acute toxicity was expected to be mild and more frequent following WPRT, whereas persistent late toxicity was infrequent, mild, and limited to a low rate of reduced sweating. Pending definitive data on the efficacy of LDRT in Dupuytren’s disease, these findings support individualized field selection guided by disease distribution, clinical judgment, and patient preference.

## Introduction

Dupuytren’s disease (DD) is a fibroproliferative disorder characterized by progressive thickening of the palmar fascia in the early stages and contractures in the later stages.^1^ It affects approximately 20% of adults aged >50 years in populations with a Northern European background.^2^ The disease progresses through proliferative and involutional phases, potentially providing a therapeutic window for preventive intervention before contracture formation.^1^ Risk factors and disease associations include age, male sex, family history, smoking, alcohol use, and metabolic comorbidity, though reported strengths of associations vary across studies.^3^^,^^4^ Low-dose radiation therapy (LDRT) has a longstanding role in nonmalignant proliferative conditions.^5^ Retrospective evidence suggests LDRT may reduce the risk of DD progression when applied during the proliferative phase, though prospective evidence is limited.^6^, ^7^, ^8^

Guidelines from both the Deutsche Gesellschaft für Radioonkologie and the Royal College of Radiologists recommend irradiating only the disease-involved area in an effort to reduce toxicity.^9^^,^^10^ However, the distribution of DD is based on clinical examination and may underestimate the extent of subclinical fascial involvement. Consequently, there is potential variability in how the optimal treatment field is defined. While more comprehensive treatment of palmar fascia could theoretically reduce the risk of untreated disease progression or field-border recurrence, it may also increase treatment-related toxicity. Given the relatively low radiation doses used in DD, the balance between treatment coverage and toxicity remains uncertain. Also, given the relatively low doses of radiation used for DD, it is feasible that additional toxicity from a more comprehensive approach may be modest. As such, there is merit in reconsidering the morbidity and efficacy of different radiation therapy treatment approaches.

The DD Evaluation of Preventative or Adjuvant Radiation Therapy (DEPART) trial randomized 404 hands from 12 Australian and Dutch centers to observation or LDRT (30 Gy in 10 fractions, split-course). Early analyses of the prevention cohort demonstrated that radiation therapy was well tolerated, with predominantly low-grade, self-limiting toxicity and no deterioration in patient-reported quality-of-life (QoL) outcomes during early follow-up. The primary efficacy endpoint of contracture prevention continues to mature.^11^ Treatment field selection was based on clinician preference, providing a unique opportunity to compare whole-palm radiation therapy (WPRT) with involved-field radiation therapy (IFRT). This substudy provides the first prospective comparison of QoL and toxicities between WPRT and IFRT for DD.

## Methods and Materials

### Trial design

The DEPART trial received approval from the Human Research Ethics Committee (2018-02-134-PVR-1) and was prospectively registered (ACTRN12618000951257). All participants gave written informed consent. Eligible participants had clinically diagnosed DD with documented progression over 6 months and <10 degrees of passive extension deficiency in any joint of the affected hand corresponding to modified Tubiana stage 0 or 0/N disease. Patients were permitted to enroll only once in the prevention cohort; therefore, each randomized unit corresponded to a single affected hand from an individual patient. From 2018 to 2024, 404 hands were randomized 1:1 to observation or LDRT, with 202 per arm. This secondary analysis compares QoL and toxicity data between WPRT and IFRT.

### Radiation therapy technique

LDRT consisted of 30 Gy in 10 fractions delivered in two 5-fraction courses with a 4- to 12-week mid-treatment break using a single 4- to 6-MeV electron beam with an appropriate bolus.^11^ WPRT covered the entire palmar surface from the wrist to the first interphalangeal joints. IFRT was adapted for clinically apparent disease with 1 to 2 cm margins. Field selection was determined by the treating radiation oncologist based on clinical assessment of disease distribution. Contemporary guidelines recommend treating the involved palmar fascia with a partial-field approach; however, this recommendation is largely based on expert consensus rather than comparative outcome data. Accordingly, the DEPART protocol permitted either WPRT or IFRT to reflect variation in contemporary practice and to allow this exploratory post hoc assessment of whether treatment field size influences toxicity or patient-reported outcomes.

### QoL and toxicity data collection

QoL assessments were planned at baseline and at 6, 12, 24, 36, 48, 60, 84, and 108 months. Because only medium-term follow-up is currently available, for the purpose of this substudy, outcomes through 36 months are reported. Three measures were used to evaluate QoL: pain on a visual analog scale (VAS), the Quick Disabilities of the Arm, Shoulder, and Hand (QuickDASH), and the Unité Rhumatologique des Affections de la Main (URAM). The pain VAS allows patients to rate their average hand pain on a scale of 0 to 10, with lower scores indicating less pain. The QuickDASH provides a score from 0 to 100 that measures upper-extremity function and symptoms; lower scores indicate better function.^12^, ^13^, ^14^ The URAM score, on a scale of 0 to 45, is a Dupuytren’s-specific QoL measure, with lower scores indicating fewer symptoms.^15^ Toxicity was systematically graded using the National Cancer Institute Common Terminology Criteria for Adverse Events version 4.03, including radiation dermatitis, dry skin, hypohidrosis, skin pain, skin atrophy, skin induration, localized edema, and arthralgia, planned to be collected at baseline, end of treatment, 30 to 37 days posttreatment, and at 6, 12, 24, 36, 48, 60, 84, and 108 months.^16^

### Statistical analysis

Descriptive data included means with SDs and 95% CIs. Within-group changes from baseline to 36 months were assessed using paired *t* tests. Between-group comparisons at each time point were conducted using independent *t* tests. For hypothesis generation and to account for multiple testing, potential statistical significance was set at *P* < .01.

## Results

### Demographics

Among 202 hands receiving LDRT, 126 (62.4%) received WPRT, and 76 (37.6%) received IFRT. Follow-up at 12 months was 95% for WPRT and 86% for IFRT; at 36 months, it was 60% and 30%, respectively. Baseline demographic and clinical characteristics were well balanced between the groups, as shown in Table 1. Both groups had similar distributions of age, sex, and hand dominance (Table E1).

**Table 1:** Baseline demographics

Characteristic	Overall (N = 202)	Whole-palm (n = 126)	Involved field (n = 76)
Age (years)	61.2 (8.7)	62.5 (7.9)	59.1 (9.5)
Sex			
Women	107 (53)	66 (52)	41 (54)
Men	95 (47)	60 (48)	35 (46)
Hand on trial			
Left	109 (54)	67 (53)	42 (55)
Right	93 (46)	59 (47)	34 (45)
Tobacco use			
Before	66 (33)	41 (33)	25 (33)
No	125 (62)	77 (61)	48 (64)
Ongoing	7 (3)	5 (4)	2 (2)
Not reported	4 (2)	3 (2)	1 (1)
Alcohol use			
No	43 (21)	26 (21)	17 (22)
Ongoing	155 (77)	97 (77)	58 (76)
Not reported	4 (2)	3 (2)	1 (1)
Peyronie’s disease (male patients)			
No	89 (94)	59 (98)	30 (86)
Yes	5 (5)	1 (2)	4 (11)
Not reported	1 (1)	0 (0)	1 (3)
Ledderhose disease			
No	159 (79)	103 (82)	56 (74)
Yes	39 (19)	20 (16)	19 (25)
Not reported	4 (2)	3 (2)	1 (1)
Family history of Dupuytren’s disease			
No	112 (55)	77 (61)	35 (46)
Yes	85 (42)	45 (36)	40 (53)
Not reported	5 (3)	4 (3)	1 (1)
Diabetes			
No	177 (88)	109 (87)	68 (90)
Yes	21 (10)	14 (11)	7 (9)
Not reported	4 (2)	3 (2)	1 (1)

Data are presented as n (%).

### QoL outcomes

**Figure 1:**
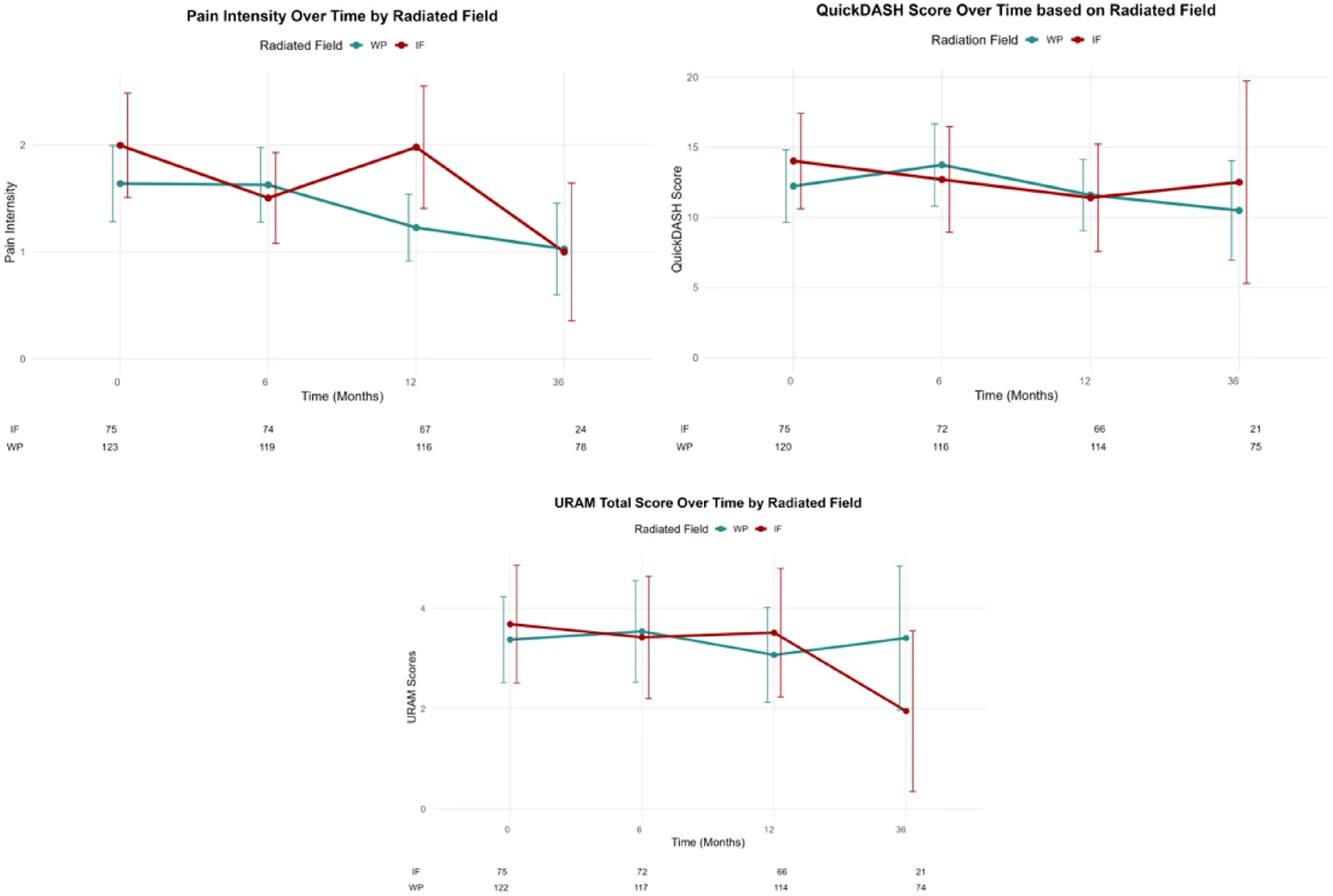
Whole-palm (WP) and involved-field (IF) radiation therapy mean pain visual analog scale, Quick Disabilities of the Arm, Shoulder, and Hand (QuickDASH), and Unité Rhumatologique des Affections de la Main (URAM) scores from baseline to 36 months.

Both groups had minimal functional impairment at baseline, reflecting early-stage disease before significant contractures. Figure 1 shows longitudinal QoL outcomes, measured by a pain intensity VAS, QuickDASH, and URAM, for hands receiving WPRT or IFRT at baseline and at 6, 12, and 36 months. Both approaches maintained hand function without treatment-related deterioration and showed stable or improved pain scores over time. The only difference in pain was at 12 months, with WPRT having slightly lower pain scores than IFRT, although the difference was not significant at the .01 level. This difference was modest, and by 36 months, pain scores converged and remained low in both field designs.

### Toxicity outcomes

**Figure 2:**
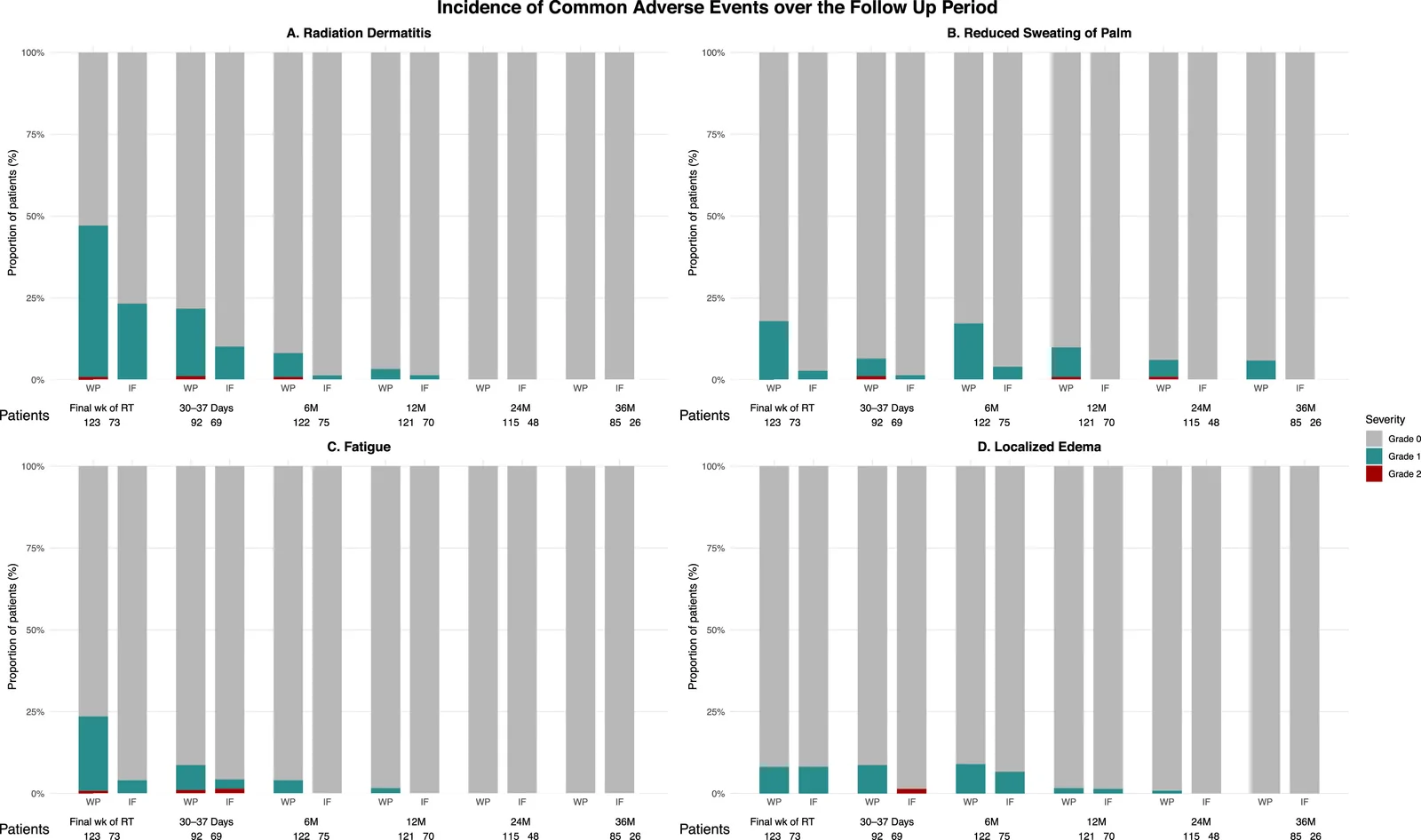
Incidence of common adverse events over the follow-up period.*Abbreviations:* IF = involved field; M = month; wk = week; RT = radiation therapy; WP = whole-palm.

LDRT was well tolerated across both treatment approaches, with all treatment-related adverse events graded per the National Cancer Institute Common Terminology Criteria for Adverse Events as grade 1 or 2, and no grade 3 or higher toxicity was observed. The incidence and severity of toxicities for each treatment group are summarized in Fig. 2. Treatment-related toxicity was more frequently reported following WPRT than IFRT, with differences most evident during treatment and early follow-up and persisting to 6 months for several toxicity domains.

Dermatitis was the most reported acute toxicity, peaking during the final week of radiation therapy and declining thereafter. Rates were higher in the WPRT cohort at all time points, with predominantly grade 1 events. Fatigue followed a similar early pattern, with peak incidence during the early posttreatment period (30-37 days) and substantial resolution by 6 months. Localized edema was observed in both cohorts and resolved early. While toxicity peaked during the final treatment week and at 30 to 37 days, several domains, particularly dermatitis and reduced sweating, remained more common in the WPRT cohort at 6 months.

Reduced sweating demonstrated a more protracted temporal course, particularly following WPRT, with low-grade symptoms persisting in a small proportion of hands at later follow-up. In contrast, reduced sweating following IFRT was uncommon and resolved after 6 months.

Across both treatment approaches, toxicity peaked during or shortly after radiation therapy, with progressive resolution over time. Persistent late toxicity was uncommon, with a <5% incidence of low-grade reduced sweating in the WPRT cohort, without associated functional impairment of the affected hands.

## Discussion

This substudy of the DEPART trial is the first to provide a direct comparison of QoL outcomes between WPRT and IFRT for DD. Over 36 months of follow-up, no sustained differences in QoL outcomes were observed between the groups. QuickDASH and URAM scores remained well below thresholds for clinically meaningful disability in both groups throughout follow-up. These results support the tolerability and favorable patient-reported QoL outcomes associated with both WPRT and IFRT in early DD. Both groups continued to report low levels of functional impairment and pain, with the 12-month pain difference modest in absolute terms. These results indicate that both radiation therapy field designs provide comparable and sustained QoL impacts. Another important consideration when selecting the treatment field size is the potential for geographic miss if treatment is limited to clinically evident nodules or cords. DD may involve broader areas of palmar fascia than clinically apparent, raising the possibility that highly localized treatment fields may fail to encompass subclinical disease. WPRT may therefore reduce the theoretical risk of field-border recurrence, although comparative prospective data addressing this question remain limited.

Although WPRT was associated with a higher incidence of acute toxicity, reflecting the greater extent of treated tissue, adverse events were overwhelmingly mild, self-limiting, and without clinically meaningful late sequelae. The observed temporal pattern of toxicity resolution is in keeping with expected tissue responses to low-dose irradiation. Cutaneous reactions, localized edema, and fatigue peaked during treatment or early follow-up and largely resolved within 6 months. Reduced sweating was more protracted following WPRT in a small proportion of patients but remained predominantly low-grade and did not result in functional impairment of the affected hands in QoL assessments.

The isolated between-group difference in the pain VAS observed at 12 months was modest in magnitude but did not persist at 36 months. It was not accompanied by differences in QuickDASH or URAM scores at any time point. In keeping with the primary DEPART analyses, this finding is most consistent with chance variation rather than a durable or clinically meaningful difference in patient-reported outcomes.

Importantly, no grade 3 or higher toxicity was observed, and persistent late toxicity was infrequent and limited to low-grade symptoms, predominantly reduced sweating. These findings support the safety of both IFRT and WPRT approaches and suggest that the modest increase in low-grade toxicity observed with WPRT should be weighed against potential therapeutic benefit. Comparisons between radiation therapy and observation arms, including QoL outcomes, have been reported in the primary DEPART analyses and will be further evaluated once the primary efficacy endpoint of contracture development (defined as a passive extensive deficiency of >20 degrees in any joint of the affected hand) is met, as well as whether field size influences this.^17^

This analysis has several important limitations. It represents a post hoc comparison with unequal group sizes, reflecting institutional practice rather than randomization, which can lead to potential selection bias. Field selection was determined by clinician assessment of disease distribution, introducing the potential for selection bias and unmeasured confounding. Patients with more extensive or multifocal disease may have been more likely to receive WPRT. Furthermore, more granular disease staging variables were not prospectively captured, limiting the ability to fully adjust for differences in disease burden between groups. Additionally, the smaller IFRT cohort with shorter follow-up limits statistical power. Follow-up is presently limited to 36 months, precluding assessment of late toxicity and long-term QoL outcomes. Despite these limitations, this analysis represents the largest prospective study to date examining radiation therapy field size in early-stage DD using validated patient-reported outcome measures. Both approaches preserved baseline function through 36 months, consistent with the objective of preventive treatment. Importantly, although toxicity measured beyond 12 months was only observed in the WPRT group, these toxicities were low grade and not associated with functional impairment. The comparison between WPRT and IFRT will become particularly informative once mature efficacy, toxicity, and QoL data from the DEPART trial are available.

Beyond field size selection in DD, these findings highlight the broader need for high-quality prospective evidence to define the role of radiation therapy in proliferative nonmalignant conditions. DD exemplifies this challenge, where clinical adoption of LDRT has occurred despite limited randomized data and ongoing variability in dose, technique, and field definition.^18^, ^19^, ^20^ Alternative dose and fractionation schedules, most commonly 21 Gy in 7 fractions without a treatment break, are used in clinical practice and may influence both toxicity and disease-control outcomes. The Radiohtherapy for Ledderhose disease study, a randomized controlled trial of radiation therapy for Ledderhose disease, provides a contemporary model for addressing these evidence gaps.^19^ It has demonstrated the feasibility of a rigorous trial design, validated patient-reported outcome measures, and long-term functional endpoints to demonstrate the benefit of radiation therapy in the management of a related condition affecting the foot. The recently published intersociety framework for radiation therapy in nonmalignant disease offers an internationally endorsed approach to developing high-quality evidence, fostering collaboration between clinicians and patients, and advancing education across nonmalignant radiation therapy indications.^5^ Together, these initiatives underscore the feasibility and importance of coordinated, methodologically robust trials to guide practice and strengthen evidence-based patient counseling in nonmalignant radiation therapy.

## Conclusions

In this post hoc comparison, both WPRT and IFRT preserved baseline hand function and demonstrated acceptable toxicity profiles through 36 months, supporting the clinical use of either approach in early-stage DD. While WPRT was associated with higher rates of low-grade toxicity, these effects were not functionally limiting, and no meaningful differences in QoL were observed. Until mature disease-control and toxicity data from the DEPART trial become available, field size selection should remain individualized, incorporating institutional experience and the desired balance among treatment comprehensiveness, toxicity, and patient preferences.^18^ Ongoing follow-up and future randomized studies, aligned with the intersociety framework, will be essential to determine whether field size should ultimately be standardized within evidence-based models of nonmalignant radiation therapy.^5^

## Disclosures

Employment/stock: GenesisCare (Renee Duvenage, Jarad M. Martin, David Christie, Joshua Sappiatzer, Katherine Neville, Sandy Sampaio, David Schlect, and Joseph Bucci). Consultancy/advisory roles: SeeTreat (Jarad M. Martin), Varian/Asellas (Jarad M. Martin), and Fidia/Novotech (Paul M.N. Werker). Research funding: GenesisCare and Varian (Jarad M. Martin). All other authors have no competing interests to disclose.

## Appendix Supplementary materials

APPENDIX-ebm-lung-sbrt_R3.docx Image, application 1

## Footnotes

Supplementary material associated with this article can be found in the online version at doi:10.1016/j.adro.2026.102083.
