# Supplementary material for: Quality of Life and Toxicities Following Whole-Palm Versus Involved-Field Radiation Therapy for Dupuytren’s Disease: A Substudy From a Randomized Trial
Source: Adv Radiat Oncol. 2026 Sep 11;11(9):102083. doi: 10.1016/j.adro.2026.102083 (PMC13576699; doi:10.1016/j.adro.2026.102083)
Supplement: Supplementary file 1 [file mmc1.docx]

**Table E1** Between-group comparisons of quality-of-life measures

| Measures | Visit | Whole-palm | Involved field | Difference (95% CI) | *P* value |
| --- | --- | --- | --- | --- | --- |
| Pain VAS |  |  |  |  |  |
|  | Baseline | 1.64 | 2.00 | −0.36 (−0.97, 0.24) | 0.24 |
|  | 6 mo | 1.63 | 1.50 | 0.13 (−0.42, 0.68) | 0.66 |
|  | 12 mo | 1.23 | 1.98 | −0.75 (−1.4, −0.10) | 0.02 |
|  | 36 mo | 1.03 | 1.03 | 0 (−0.73, 0.79) | 0.94 |
| QuickDASH |  |  |  |  |  |
|  | Baseline | 12.22 | 14.01 | −1.79 (−6.04, 2.46) | 0.41 |
|  | 6 mo | 13.78 | 12.69 | 1.09 (−3.70, 5.78) | 0.67 |
|  | 12 mo | 11.58 | 11.39 | 0.19 (−4.37, 4.75) | 0.93 |
|  | 36 mo | 10.48 | 12.50 | −2.02 (−9.94, 5.91) | 0.61 |
| URAM |  |  |  |  |  |
|  | Baseline | 3.38 | 3.68 | −0.30 (1.75, 1.14) | 0.67 |
|  | 6 mo | 3.54 | 3.42 | 0.12 (−1.45, 1.69) | 0.88 |
|  | 12 mo | 3.07 | 3.52 | −0.45 (−2.02, 1.14) | 0.58 |
|  | 36 mo | 3.41 | 1.95 | 1.46 (−0.65, 3.56) | 0.17 |

*Abbreviations:* QuickDASH = Quick Disabilities of the Arm, Shoulder, and Hand; URAM = Unité Rhumatologique des Affections de la Main; VAS = visual analog scale.
